# Uptake of L-cystine via an ABC transporter contributes defense of oxidative stress in the L-cystine export-dependent manner in *Escherichia coli*

**DOI:** 10.1371/journal.pone.0120619

**Published:** 2015-04-02

**Authors:** Iwao Ohtsu, Yusuke Kawano, Marina Suzuki, Susumu Morigasaki, Kyohei Saiki, Shunsuke Yamazaki, Gen Nonaka, Hiroshi Takagi

**Affiliations:** 1 Graduate School of Biological Sciences, Nara Institute of Science and Technology, Ikoma, Nara, Japan; 2 Research Institute for Bioscience Products and Fine Chemicals, Ajinomoto Company, Kawasaki, Kanagawa, Japan; Arizona State University, UNITED STATES

## Abstract

Intracellular thiols like L-cystine and L-cystine play a critical role in the regulation of cellular processes. Here we show that *Escherichia coli* has two L-cystine transporters, the symporter YdjN and the ATP-binding cassette importer FliY-YecSC. These proteins import L-cystine, an oxidized product of L-cystine from the periplasm to the cytoplasm. The symporter YdjN, which is expected to be a new member of the L-cystine regulon, is a low affinity L-cystine transporter (*K*_m_ = 1.1 μM) that is mainly involved in L-cystine uptake from outside as a nutrient. *E*. *coli* has only two L-cystine importers because Δ*ydjN*Δ*yecS* mutant cells are not capable of growing in the minimal medium containing L-cystine as a sole sulfur source. Another protein YecSC is the FliY-dependent L-cystine transporter that functions cooperatively with the L-cystine transporter YdeD, which exports L-cystine as reducing equivalents from the cytoplasm to the periplasm, to prevent *E*. *coli* cells from oxidative stress. The exported L-cystine can reduce the periplasmic hydrogen peroxide to water, and then generated L-cystine is imported back into the cytoplasm via the ATP-binding cassette transporter YecSC with a high affinity to L-cystine (*K*_m_ = 110 nM) in a manner dependent on FliY, the periplasmic L-cystine-binding protein. The double disruption of *ydeD* and *fliY* increased cellular levels of lipid peroxides. From these findings, we propose that the hydrogen peroxide-inducible L-cystine/L-cystine shuttle system plays a role of detoxification of hydrogen peroxide before lipid peroxidation occurs, and then might specific prevent damage to membrane lipids.

## Introduction

Excessive reactive oxygen species (ROS) are cytotoxic molecules, which result in the oxidation of DNA, proteins and cellular membrane lipids [1, 2]. Inside the cell, it generates from their respiration chain and various redox reactions which results in the exchange of one or two electrons to molecular oxygen [3]. Most organisms acquire various ROS scavenging strategies for their survival and conservation of the species. For example, *Escherichia coli* possesses ROS-scavenging enzymes such as superoxide dismutase (2O_2_^–^ + 2H^+^ → O_2_ + H_2_O_2_), catalase (2H_2_O_2_ → O_2_ + 2H_2_O), and peroxidase (ROOR' + 2e^–^ + 2H^+^ → ROH + R'OH). Superoxide dismutases exist both in the cytoplasm (SodA and SodB) and the periplasm (SodC), whereas catalase is present only in the cytoplasm (KatE) but not in the periplasm. Unlike the cytoplasm, the mechanism underlying the process for scavenging periplasmic H_2_O_2_ generated from the SodC reaction has remained unclear [4].

Recently, we found that the *E*. *coli* mutants lacking the l-cysteine (Cys) exporter YdeD or the l-cystine (CySS) binding protein FliY increased sensitivity to H_2_O_2_ [5]. It was also shown that FliY is involved in the uptake of CySS from the periplasm to the cytoplasm. Moreover, the expressions of the *ydeD* and *fliY* genes were dramatically increased upon treatment of the cells with H_2_O_2_ [5]. From these findings, we proposed that *E*. *coli* removes the periplasmic H_2_O_2_ using Cys supplied to the periplasm from the cytoplasm by “Cys/CySS shuttle system” [5]. In this system, however, it remains questionable i) whether Δ*ydeD* mutant cells actually accumulate H_2_O_2_ in the periplasm, ii) what is the FliY-dependent CySS importer, and iii) whether the CySS importer cooperatively operates with YdeD. In addition to them, the question arises as to whether endogenous Cys *de novo* synthesized from inorganic sulfur source serves the function of Cys /CySS shuttle system.

Thus, we speculated that not only a Cys exporter such as YdeD but also a CySS importer such as the FliY-dependent protein is critical for protecting cells from oxidative stress into the periplasm due to quality control of the membrane. These considerations have led us to study the role of CySS importers in *E*. *coli*. In this report, we identified two different CySS importers that indeed function against H_2_O_2_ stress in *E*. *coli*. We also showed evidence that these CySS importers cooperatively function with the Cys exporter YdeD to scavenge the periplasmic H_2_O_2_. Furthermore, our data indicate that endogenous Cys *de novo* synthesized from inorganic sulfur source is utilized to eliminate the periplasmic H_2_O_2_. Thus, we propose that the hydrogen peroxide-inducible Cys/CySS shuttle system has an important role for quality control of the plasma membrane in *E*. *coli* cells.

## Materials and Methods

### Bacterial strains and oligonucleotides

Gene cloning, DNA manipulation and the transformation of *E*. *coli* strains were performed according to the standard methods [6]. *E*. *coli* wild-type strain, BW25113 and their derivatives (single gene deletion mutants) were supplied by the National Bio Resource Project (NBRP, Keio collection). An Hpx^—^mutant strain [7] was kindly provided by James A. Imlay (University of Illinois, Illinois, USA). Double gene deletion mutants were constructed from the single deletion mutants as a parental strain. We used the same method as that of Baba *et al*. [8], including primer sequences. Almost all the open-reading frame coding regions were replaced with an antibiotics resistant cassette flanked by FLP recognition target sites by using a one-step method for inactivation of chromosomal genes. If needed, the antibiotics resistant cassette (kanamycin or chloramphenicol) was removed according to the reference [8]. The constructed strains are listed in S1 Table.

### Media and Growth Conditions

*E*. *coli* was cultivated with Luria-Bertani (LB) complete medium or an M9_S0_ minimal medium (6 g/L Na_2_HPO_4_, 3 g/L KH_2_PO_4_, 0.5 g/L NaCl, 1 g/L NH_4_Cl, 4 g/L glucose, 1 mM MgCl_2_, and 0.05% (w/v) thiamine-HCl) supplementing an indicated sulfur source as a sole sulfur source. The liquid cultures were performed with shaking. Growth of cultures was monitored by measuring of the optical density at 660 nm (OD_660_). We assumed a reading for OD_660_ of 1 corresponded to 5.0 × 10^9^ cells ml^–1^. When appropriate, antibiotics were added at 50 μg/ml (for kanamycin), 30 μg/ml (for chloramphenicol). For solid media, 1.5% agar was added.

### l-Cystine uptake assays

The basic method for CySS uptake assays was as previously described [5]. Briefly, cells grown to mid-exponential phase were harvested by centrifugation, washed three times with cold KPM solution (10 mM MgSO_4_, 0.1 M K_2_HPO_4_; pH was adjusted to 6.5 with H_3_PO_4_), and resuspended in cold KPM solution (OD_660_ = 0.4). Portions of the cell suspension (6.5 ml each) were energized, by the addition of 60 μl of 40% D-glucose, followed by incubation for 10 min at 37°C. The CySS uptake assay was initiated by the addition of indicated concentrations of CySS containing radiolabeled l-[^14^C]-CySS (291.3 mCi/mmol). Following the incubation of the cells at room temperature for 10 min, the cells were collected by filtration through a GF/C filter (Whatman), and washed three times with KPM solution. Then, the radioactivity derived from ^14^C incorporated into the cells on the filter was determined by liquid scintillation counter LSC-7200 (Beckman). The calculated cystine uptake amount was used for cystine uptake rate (pmoles [10^9^ cells]^–1^ min^–1^). The 10 min incubation for cystine uptake was appropriate to evaluate the initial rate because the uptake rate was proportional at least until 20 min in all conditions performed. To determine the kinetic parameters, plot of CySS uptake rate versus CySS concentrations were performed, and the apparent *V*_max_ and *K*_m_ were calculated by using a Hanes-Woolf plot.

### Real-time quantitative polymerase chain reaction analysis

Primers were designed using the Primer Express (Applied Biosystems, Foster City, CA, USA) and listed in S2 Table. The procedure of mRNA extraction, reverse transcription, and real-time PCR was as previously described [5]. The mRNA level of each gene was normalized to that of *rrsH* (internal standard) in the same sample.

### Staining of periplasmic H_2_O_2_ and fluorescence microscopy

Cells were grown to mid-exponential phase in LB medium, harvested by centrifugation, washed and resuspended with dH_2_O. After addition of 1 mM pentafluorobenzenesulfonyl-2',7'-difluorofluorescein (cell-impermeant fluorescent H_2_O_2_ probe; BES-H_2_O_2_, Wako), the cell suspension was incubated for 30 min at room temperature. After washed with dH_2_O, the stained cells were resuspended with dH_2_O, and subjected to fluorescence microscopic analyses. For the visualization, cells were viewed under an Axiovert 200 M microscope (Carl Zeiss) with a 100× oil immersion objective. The probe was excited by light at 485 nm and the fluorescence emission at 530 nm was observed. The images were captured with an AxioCam MRm CCD camera (Carl Zeiss). The observed staining pattern was categorized into three types; (i) cells not stained at all, (ii) the cells whose cytoplasm was stained, (iii) the cells whose periplasm was stained (the representative cell was indicated by arrow and shown by the close-up image). Ratio of these types of cells were shown in the graph. To determine the ratio, more than 100 cells were investigated in each strain. The phase-contrast images were also photographed in the same sample to count cells.

### Analysis of lipid peroxides of cellular membrane

To estimate the extent of lipid peroxides of cellular membrane in *E*. *coli*, its byproduct, malondialdehyde (MDA), was measured as thiobarbituric acid reactive substances (TBARS) [9, 10]. Cells were grown in LB medium with 1 mM or 75 μM H_2_O_2_, or M9 medium (1 mM MgSO_4_) without H_2_O_2_, and then 10 ml of cells (10^8^ cells/ml) culture were harvested by centrifugation at indicated time. TBARS solution (0.01% 3,5-dibutyl-4-hydroxytoluene, 20% trichloro acetic acid, and 0.65% thiobarbituric acid after dissolution by 100°C incubation) was added to the cells, which was treated at 100°C for 25 min. Then, the resulting solution was incubated at room temperature for 5 min, on ice for 5 min, and then centrifuged for 5 min at 8,800 rpm. The supernatant was subjected to measurement of absorbance at 532 nm. The TBARS concentration was calculated from standard curve using authentic sample of MDA (0–50 μM). For blank measurement, duplicated cell sample was identically treated with TBARS solution that is absent of thiobarbituric acid. The net TBARS concentration was represented as MDA equivalent after normalization by cell number.

## Results

### Putative cystine importers in *E*. *coli*

**Fig 1:**
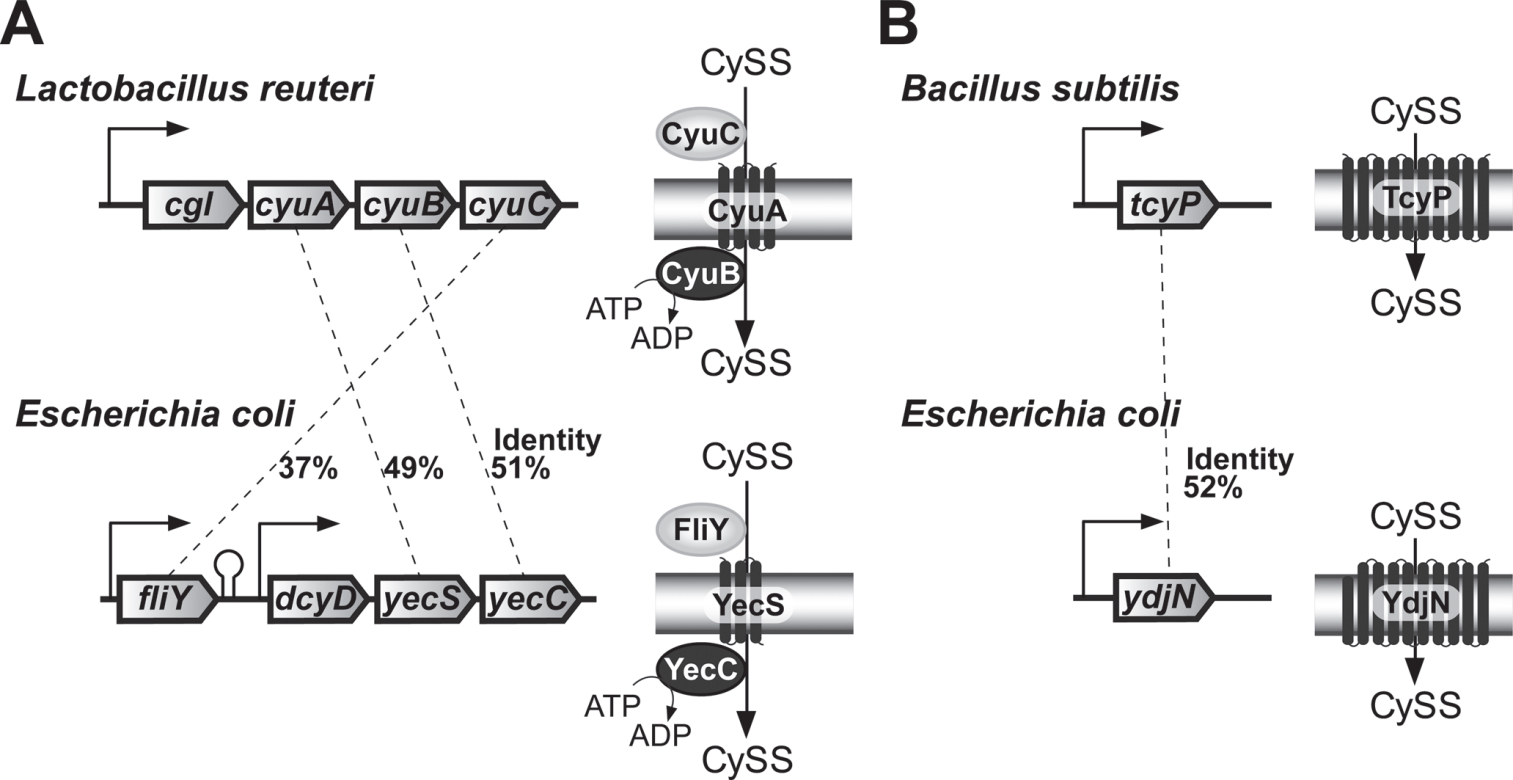
Putative CySS importer genes in *E*. *coli*. (A) ABC type CySS importer gene operon and the encoded protein constitution on the cytoplasmic membrane in *Lactobacillus reuteri* and the homologous counterpart in *E*. *coli*. (B) Symporter type CySS importer gene and the encoded protein constitution on the cytoplasmic membrane in *Bacillus subtilis* and the homologous counterpart in *E*. *coli*. The identities in amino acid sequences of homologous genes are indicated. CySS, cystine.

Some CySS importers known in bacteria are mainly classified into two types. One belongs to the ATP-binding cassette (ABC) transporter family. *Lactobacillus reutei* BR11 possesses the *cyuABC* operon, which is involved in the CySS-mediated oxidative defense (Fig. 1A) [11]. The inner membrane-spanning protein CyuA, the cytoplasmic ATP-binding protein CyuB, and the periplasmic CySS-binding protein CyuC form a complex on the cytoplasmic membrane. *Bacillus subtilis* also harbors three CySS importer systems that include two ABC transporters, TcyABC and TcyJKLMN, and another symporter TcyP [12]. In comparison to these known proteins, the FliY protein, which contributes to the CySS uptake in *E*. *coli*, exhibits sequence identities to CyuC, TcyA, TcyJ, and TcyK (30–40%, Fig. 1A). In *E*. *coli*, the *fliY* gene is unlikely to form an operon structure because probable terminator is located at its downstream. However, the putative *dcyD*-*yecSC* operon locates at the immediate downstream of *fliY* (Fig. 1A). The YecS and YecC proteins exhibit significant similarities in amino acid sequences to CyuA (49%) and CyuB (51%), respectively. YecC harbors the Walker A, Walker B, and ABC signature motifs required for ATPase activity, which is highly conservative to CyuB (S1 Fig.). These facts suggest that YecSC is a FliY-dependent CySS importer (FliY-YecSC) in *E*. *coli*. The *E*. *coli dcyD* (PLP-dependent d-cysteine desulfhydrase) gene is additionally present in the *dcyD*-*yecSC* operon and thus might work coincidentally with YecSC, although the counterpart gene is absent in CySS importer operon in *L*. *reutei* (Fig. 1A). The cystine, which is imported into the cytoplasm, might be immediately degraded to ammonium, pyruvate, and hydrogen sulfide by DcyD.

Another type of CySS importer belongs to the dicarboxylate/amino acid:cation (Na^+^ and/or H^+^) symporter family, which is included in the SLC (Solute Carrier) superfamily. The TcyP protein is a CySS importer of this kind in *B*. *subtilis* [12]. Based on a homology search, the *E*. *coli* YdjN, which is highly homologous (52% identity) to the *B*. *subtilis* TcyP, was predicted to have 10 transmembrane domains, whose C-teriminal amino acid locates in the periplasm (Fig. 1B). YdjN is thus another candidate for the CySS importer in *E*. *coli*.

### Mutant lacking both YdjN and YecS cannot grow in the medium containing CySS as a sole sulfur source

**Fig 2:**
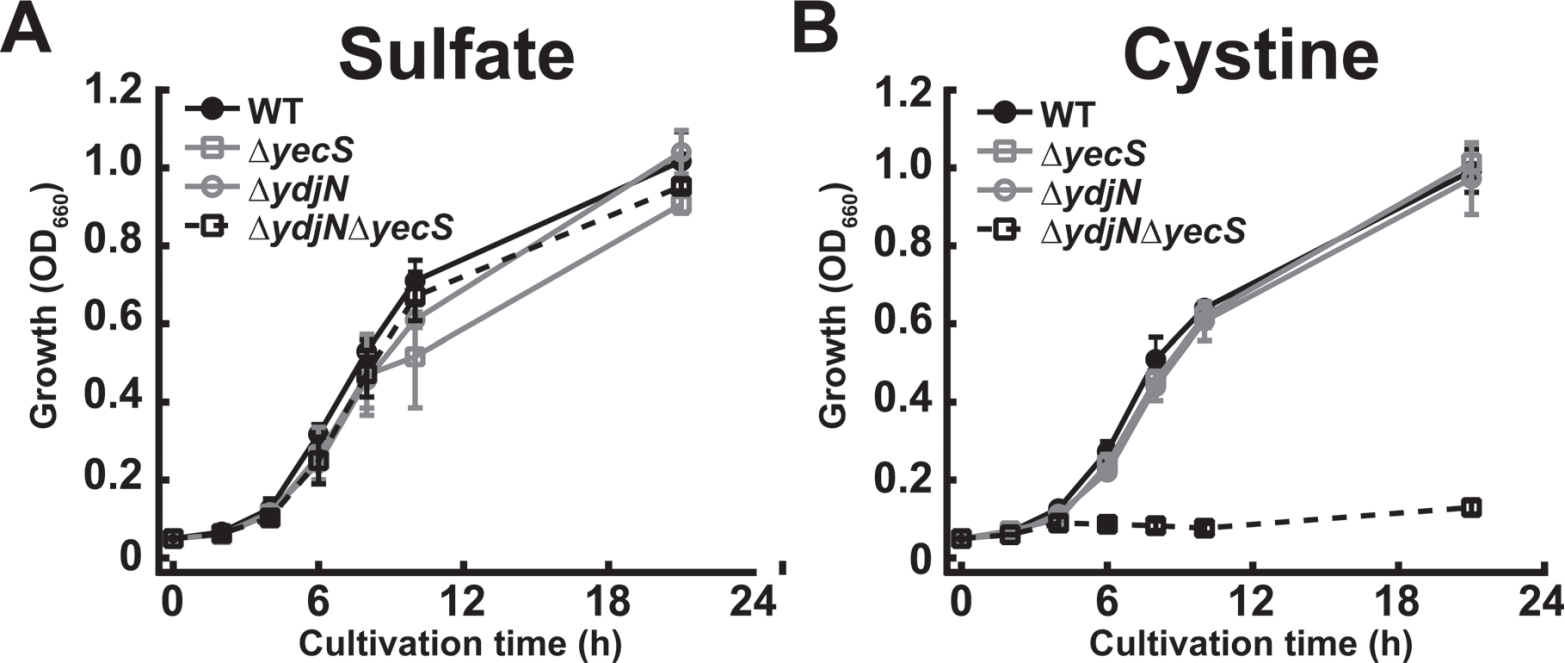
*E*. *coli* possesses two CySS importers of FliY-YecSC and YdjN. Growth of the CySS importer gene disruptants was monitored in M9 medium containing sulfate (A) or CySS (B) as a sole sulfur source.

To evaluate the relationship between the putative CySS importers and CySS utilization in growth, the wild-type, Δ*yecS*, Δ*ydjN*, and Δ*ydjN*Δ*yecS* mutant cells were grown in M9 medium containing 2 mM MgSO_4_ (Fig. 2A) or 1 mM CySS (Fig. 2B) as a sole sulfur source. All mutants were capable of growing in M9 medium containing sulfate as a sole sulfur source as well as the wild-type strain. By contrast, in the presence of CySS as a sole sulfur source, Δ*yecS* and Δ*ydjN* normally grew as well as the wild-type, whereas the growth of Δ*ydjN*Δ*yecS* was completely abolished. These results indicate that *ydjN* and *yecS* are essential for growth utilizing CySS as a sole sulfur source, suggesting that *E*. *coli* possesses two different CySS importers, FliY-YecSC and YdjN.

### FliY-YecSC and YdjN are cystine importers with distinct characteristics

**Fig 3:**
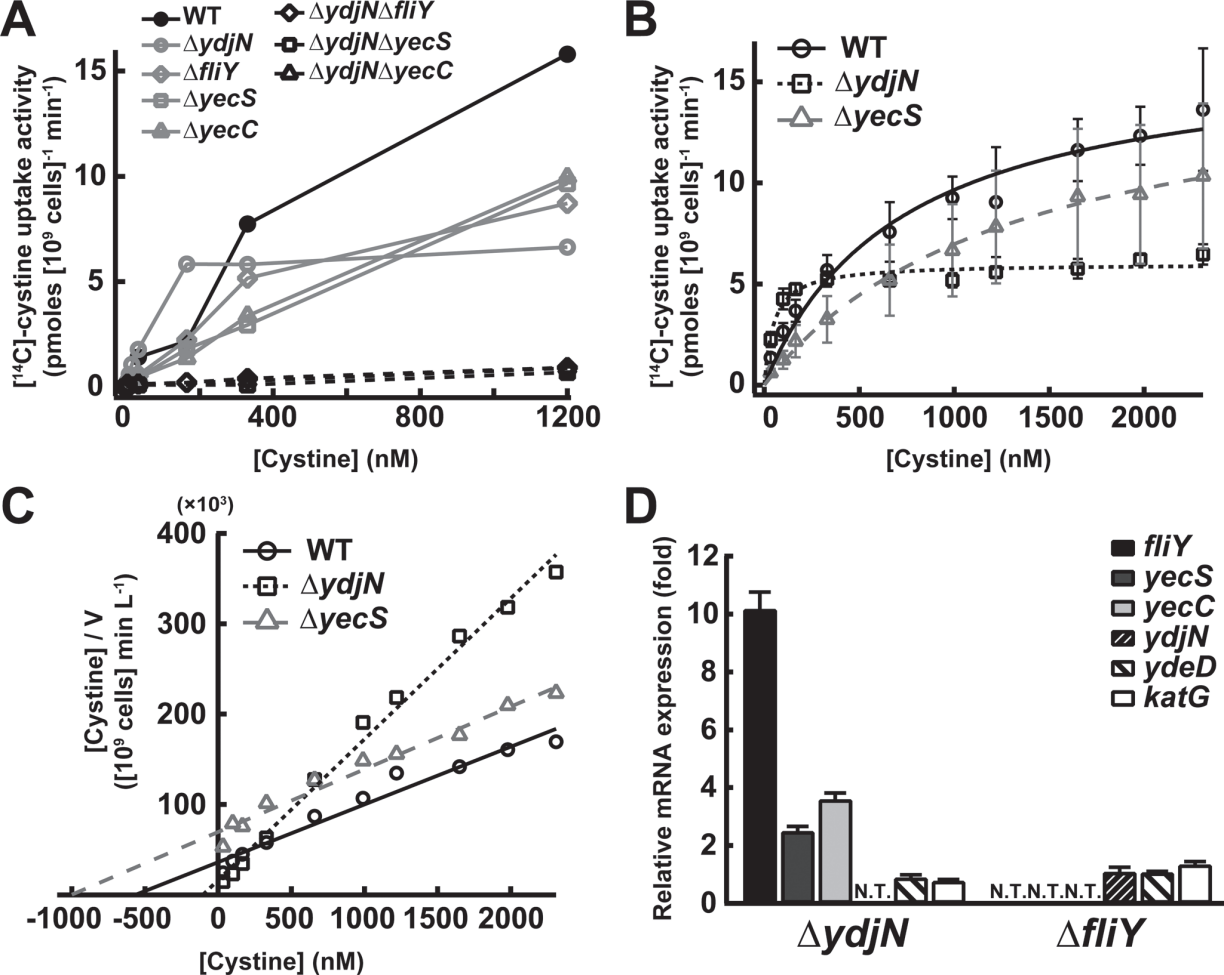
FliY-YecSC and YdjN are CySS importers with distinct properties. CySS uptake activity (initial rate) in the putative CySS importer gene disruptants (A) at various CySS concentrations and (B) in broader range of CySS concentrations to determine its kinetic parameters. Indicated concentrations of CySS containing radiolabeled [^14^C]-CySS was added to cells in assay buffer. After incubation for CySS uptake, cells were collected, and the amount of imported [^14^C]-CySS was detected by scintillation counter. (C) Hanes-Woolf plot of data shown in (B). (D) Induction of mRNA levels of CySS uptake and Cys transport genes by disruption of CySS importer gene. Cells of the exponential phase cultivated in LB medium (normal condition) were used for RT-PCR analysis. The catalase gene (*katG*) was also examined to judge the occurrence of oxidative stress by the gene disruption. The levels represent ratios compared to those of the same genes in wild-type cells.

To examine whether FliY-YecSC and YdjN are the CySS importer, we measured the uptake rate of l-[^14^C]-CySS in the Δ*fliY*, Δ*yecS*, Δ*yecC*, Δ*ydjN*, Δ*ydjN*Δ*fliY*, Δ*ydjN*Δ*yecS*, and Δ*ydjN*Δ*yecC* mutants at concentrations of 3.3–1,200 nM (Fig. 3A). As CySS concentration increased, the uptake rate of CySS in wild-type strain also increased, and reached ~15 pmol (10^9^ cells)^–1^ min^–1^ at 1,200 nM CySS. In the Δ*fliY*, Δ*yecS*, Δ*yecC*, or Δ*ydjN* mutants, the uptake rate significantly decreased to 45, 39, 37, or 58% of that in wild-type strain, respectively, at 1,200 nM CySS. Of the mutants, Δ*fliY*, Δ*yecS*, and Δ*yecC* displayed the same manner of CySS import, although only Δ*ydjN* mutant showed a different pattern. Moreover, three types of double disruption, *ydjN* plus *fliY*, *yecS*, or *yecC*, abolished the CySS import. These results suggest that FliY, YecS, and YecC function in the same module as the CySS importer of FliY-YecSC, and that *E*. *coli* conclusively possesses only two CySS importers, FliY-YecSC and YdjN.

To determine the kinetic properties of YdjN and FliY-YecSC, Δ*yecS* and Δ*ydjN* mutant, respectively, were measured CySS uptake activities together with wild-type in broader range of CySS concentrations. The plots of obtained data (CySS uptake rate versus CySS concentration) followed typical Michaelis-Menten kinetics (Fig. 3B). The Hanes-Woolf plots (Fig. 3C) showed each apparent *K*_m_ and *V*_max_ value for CySS uptake (Table 1). Interestingly, these two importers displayed quite different characteristics: FliY-YecSC, higher affinity with lower activity; YdjN, lower affinity with higher activity.

**Table 1:** Kinetic parameters of cystine importer.

**Cystine importer (strain used)**	***K***_m_ **(nM)**	***V***_max_ **(pmol [10** ^9^ **cells]** ^−1^ **min** ^−1^ **)**
**FliY-YecSC and YdjN (wild-type)**	496 ± 111	14.4 ± 4.61
**FliY-YecSC (Δ*ydjN*)**	106 ± 13.6	6.41 ± 0.700
**YdjN (Δy*ecS*)**	1061 ± 313	14.7 ± 9.67

Apparent *K*_m_ and *V*_max_ values were determined by Hanes-Woolf plots shown in Fig. 3C. The assays were performed thrice, and the average value and standard deviations are shown.

Curiously, at the range of low CySS concentrations (0–250 nM), the CySS uptake rate of the Δ*ydjN* mutant was significantly higher than that in the wild-type strain (Fig. 3 A and B). Thus, expression levels of the *fliY*-*yecSC* genes were validated by quantitative real-time (qRT) PCR in Δ*ydjN* mutant cells (Fig. 3D). The expressions of *fliY*, *yecS*, and *yecC* were 10-, 3-, and 4-fold induced, respectively, relative to those in wild-type cells, although *ydeD* (cysteine transporter) and *katG* (cytoplasmic catalase) were not induced at all. This induction might be caused by complementary effect of defect of YdjN function, and is the possible reason for the higher CySS uptake at low concentrations in Δ*ydjN* mutant. These results implicate that the CySS import ability of FliY-YecSC might be smaller than that shown here using Δ*ydjN*. On the other hand, the mRNA level of *ydjN* remains unchanged in Δ*fliY*, suggesting that YdjN functions predominantly as a nutrient uptake player under normal conditions.

### Cystine importer contributes to hydrogen peroxide tolerance in *E*. *coli*

**Fig 4:**
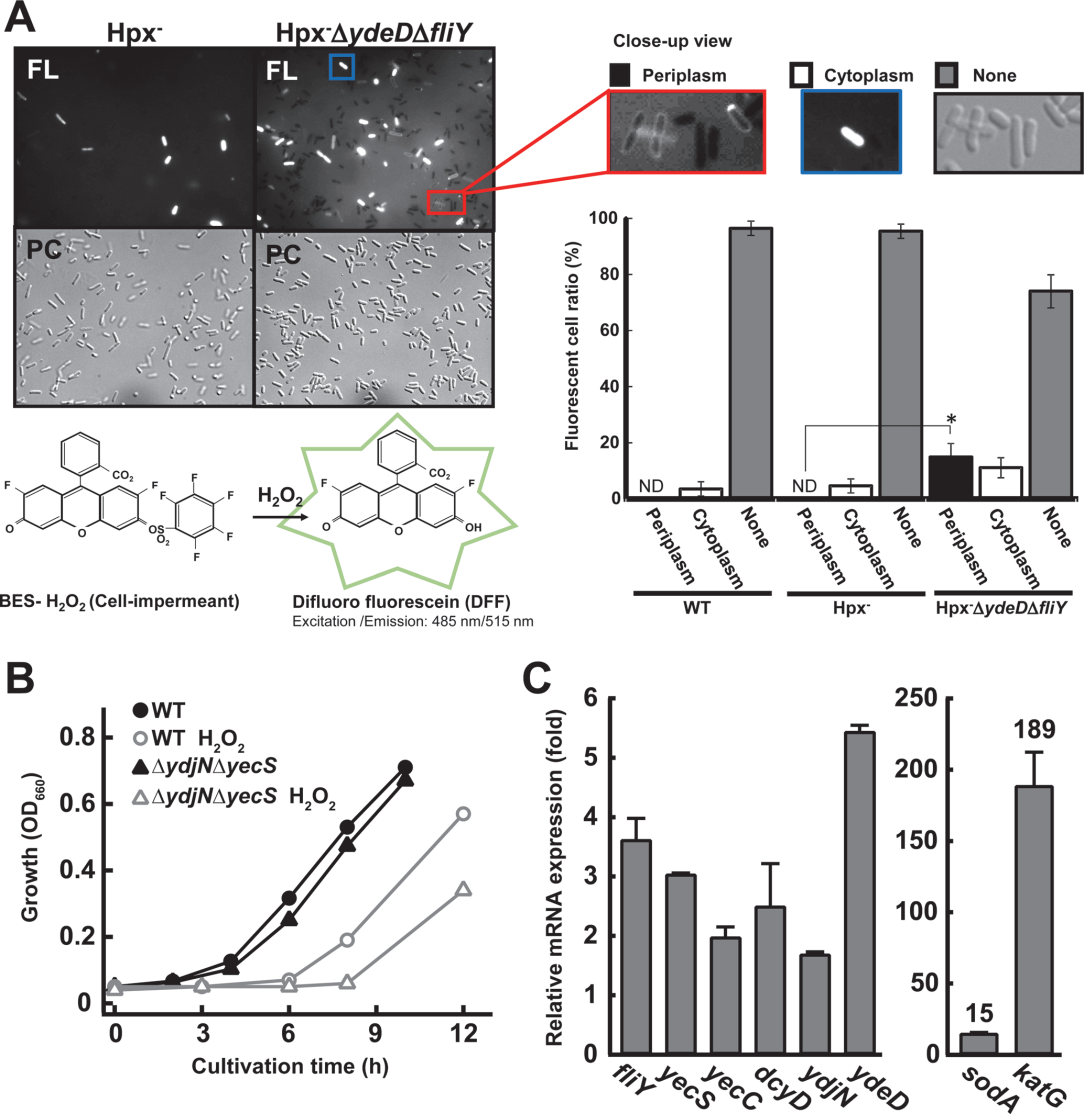
CySS importers play a role of physiological function for tolerance to H_2_O_2_ stress. (A) Accumulation of H_2_O_2_ in the periplasm by lacking the Cys/CySS shuttle system. Cells grown under normal conditions were stained with a membrane-impermeant fluorescent probe (BES-H_2_O_2_). This probe can detect cell-derived H_2_O_2_ with higher selectively than 2’,7’-Dichlorofluorescin (DCFH) and its analogues [13], and then cells were photographed using fluorescence microscopy (FL) and phase-contrast microscopy (PC). Three types of the cells were observed in fluorescence microscopy; nothing was stained (Gray bars), the cytoplasm (White bars) and the periplasm (black bar) was stained. The red box and blue box are shown by the close-up image. The percentages of the cells showing each localization pattern were calculated from 300 to 400 cells. White and black bars represent the percentages of the cells in which H_2_O_2_ is accumulated in the cytoplasm (blue box) and the periplasm (red box), respectively. * Significantly different at p<0.05. ND means “not detacted”. (B) Impairment of Cys/CySS shuttle system gives rise to prolongation of the lag-phase in growth. Growth of the CySS importer gene disruptants was monitored in M9 medium containing SO_4_^2-^ as a sole sulfur source in the presence or absence of H_2_O_2_. (C) H_2_O_2_ induction of the CySS importer genes and Cys transporter gene expression. The expression was measured by RT-PCR using cells of the exponential phase cultivated in LB medium (normal condition). The superoxide dismutase gene (*sodA*) and the catalase gene (*katG*) were also examined to confirm the occurrence of oxidative stress by addition of H_2_O_2_. The levels represent ratios compared to those of the same genes in wild-type cells.

We previously showed that Cys transport by YdeD to the periplasm and FliY-dependent CySS uptake contribute to H_2_O_2_ tolerance [5]. However, it is still unknown whether H_2_O_2_ accumulation and its scavenging by Cys/CySS shuttle system occurred in the periplasm. To present the direct evidence, periplasmic H_2_O_2_ was visualized using the H_2_O_2_ specific fluorescence probe, benzenesulfonyl (BES) chloride, which is the membrane-impermeable, under fluorescence microscopy (http://www.wako-chem.co.jp/english/labchem/product/life/BES-H2O2/index.htm) [13–15]. To locally and precisely detect periplasmic H_2_O_2_ and its specific scavenging by Cys/CySS shuttle system, Hpx^—^mutant, which has very little H_2_O_2_-scavenging activity because of lacking alkylhydroperoxide reductase (AhpCF) and two catalases (KatE and KatG) [7] was utilized as control strain. As shown in Fig. 4A, in Hpx^–^, ~95% cells were not stained, the other ~5% cells were cytoplasmically stained, and none of the cells were periplasmically stained. In Hpx^–^Δ*ydeD*Δ*fliY*, namely Cys/CySS shuttle system deficient strain, ~74% cells were not stained, ~15% cells were cytoplasmically stained, and ~11% cells were remarkably periplasmically stained. Not stained cells might include originally stained but the probe was diffused during washing after stain. Cytoplasmically stained cells might be cells with weak or disrupted cytoplasmic membrane, and allowed entering of the probe, because the probe was membrane-impermeable. Therefore, the presence of periplasmically stained cells with the H_2_O_2_ probe only in Hpx^–^Δ*ydeD*Δ*fliY* means that Cys/CySS shuttle system specifically play a role for periplasmic H_2_O_2_ scavenging.

To examine whether identified CySS importers are member of Cys/CySS shuttle system and responsible for H_2_O_2_ tolerance, Δ*ydjN*Δ*yecS* mutant cells were grown in M9 medium containing sulfate as a sole sulfur source in the presence or absence of H_2_O_2_ stress (Fig. 4B). Without H_2_O_2_ stress, wild-type and Δ*ydjN*Δ*yecS* mutant strains similarly exhibited normal growth. Under H_2_O_2_ stress conditions, the wild-type strain exhibited longer lag-phase, although the growth rate in the log-phase was almost identical. This result indicates that cells cannot transit from the lag-phase to the log-phase until suitable acclimation occurred in cellular response to H_2_O_2_. Likewise, the Δ*ydjN*Δ*yecS* mutant exhibited similar profile to the wild-type strain, but the prolongation of lag-phase by H_2_O_2_ was much longer than that of the wild-type strain. This result means that CySS importers contribute to H_2_O_2_ tolerance. Notably, because the medium contains sulfate as a sole sulfur source, de novo synthesized Cys might confer H_2_O_2_ tolerance via the CySS importers on *E*. *coli* cells.

### Induction of cystine importers by H_2_O_2_

To elucidate whether this system is inducible in response to H_2_O_2_ stress, we quantified the expression levels of Cys/CySS shuttle system-related genes in the presence or absence of H_2_O_2_ (Fig. 4C). H_2_O_2_ was shown to constitutively induce the known H_2_O_2_ responsive genes, *sodA* and *katG* [16]. Also, H_2_O_2_ relatively induced *ydeD*, *fliY*, *yecS*, *yecC* by 3.5-, 2-, 2.5-, 2-fold, respectively, and *dcyD* that probably forms an operon with *yecS* and *yecC* (Fig. 1A) by 2.5-fold. In contrast, H_2_O_2_ induction was relatively lower in the *ydjN* mutant (1.5-fold).

### Cysteine/cystine shuttle system contributed by cystine importer protects membrane lipids from H_2_O_2_

**Fig 5:**
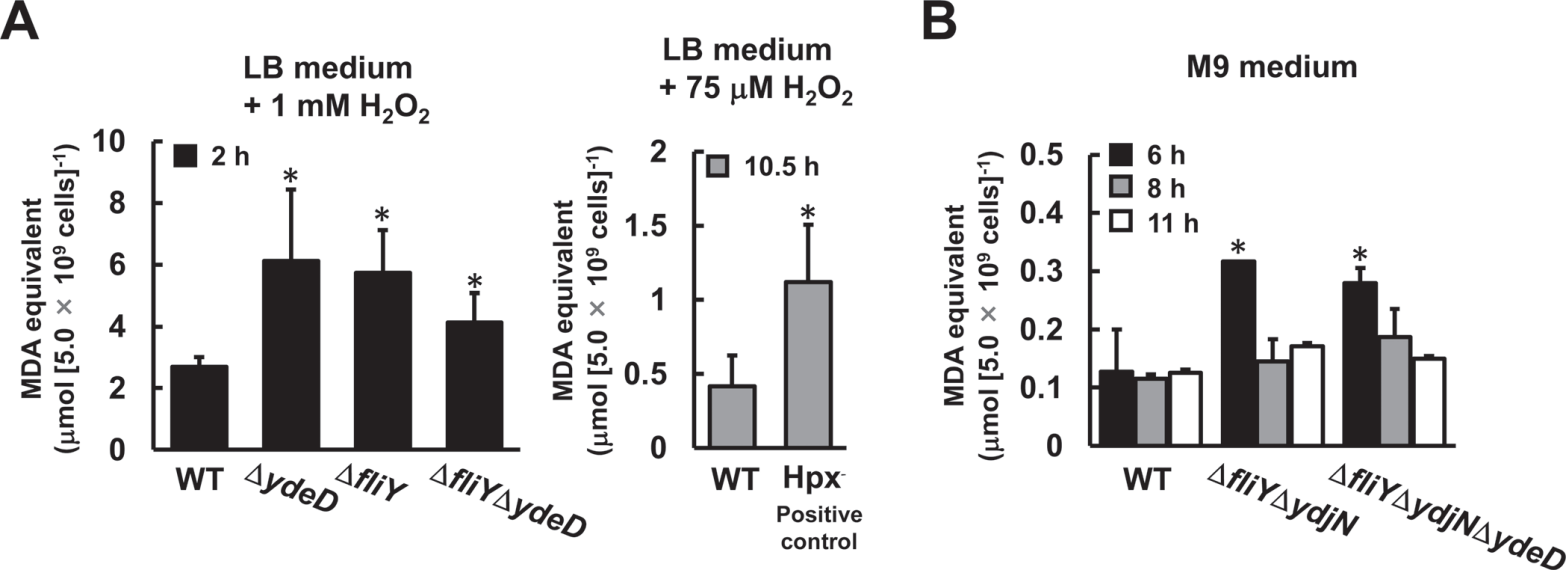
Membrane lipid peroxidation in the gene disruptants involved in Cys/CySS shuttle system. MDA generating from lipid peroxide was colorimetrically quantitated using TBA (see Materials and Methods). The cells in log-phase were diluted to OD_660_ = 0.01 and cultured at 37°C with vigorous shaking: (A) LB medium with 1 mM H_2_O_2_ for 2 h (left panel) and with 75 μM H_2_O_2_ for 10.5 h (right panel), and (B) M9 medium containing 1 mM sulfate as a sole sulfur source for indicated time. The MDA equivalent values are represented after normalization by cell number as means±SD. * Significantly different from the value of WT at the same time point at p<0.05.

In general, H_2_O_2_ causes oxidation and functional defects of lipids, resulting in cell damage. To investigate the functional effect of Cys/CySS shuttle system on H_2_O_2_ tolerance, we estimated the peroxidation extent of cellular membrane lipids. To this end, the degraded end product of lipid peroxides, malondialdehyde (MDA), was determined using thiobarbituric acid [9, 10] (Fig. 5A). After cultivation at 37°C for 2 h in the presence of 1 mM H_2_O_2_, MDA levels of the Δ*ydeD*, Δ*fliY* mutants were equally higher (2.3- and 2.1-fold, respectively) than that of the wild-type strain. Further, the MDA level of the double gene disruption, Δ*ydeD*Δ*fliY*, did not exhibit the additive effect of those of each single gene mutant (1.6-fold). This result suggests that YdeD and FliY-YecSC cooperatively contributes to the defense of membrane lipids from H_2_O_2_ stress. Using the same procedure, higher level of MDA in H_2_O_2_-treated Hpx^—^cells, whose H_2_O_2_-scavenging activity is very little (positive control), than that in WT cells was consistently detected (Fig. 5A), suggesting our method was working.

As shown in Fig. 4A, H_2_O_2_ usually generates in the periplasm and scavenged by Cys/CySS shuttle system even under normal conditions. Thus, we examined the protective function of Cys/CySS shuttle system under normal conditions (Fig. 5B). At the early log-phase (6 h), the cellular MDA level of Δ*fliY*Δ*ydjN* and Δ*fliY*Δ*ydjN*Δ*ydeD* were both 3-fold higher than that of wild-type cells (Fig. 5B), but there were significant differences between the mutants. In contrast, at the mid-log phase (8 h and 11 h), an increase observed at 6 h in the mutants almost reduced to the level of wild-type cells, suggesting that Cys/CySS shuttle system is functional in the early-log phase. Here, we also determined an intracellular protein carbonyl content by using 2, 4-dinitrophenylhydrazine. An increase of protein carbonyl content prepared from whole cells of these mutants cannot observed (data not shown). This result also means that both of CySS import and Cys export is essential for the function of membrane protection by Cys/CySS shuttle system even under normal conditions. In other words, the Cys export and CySS import cooperate for the function of Cys/CySS shuttle system.

## Discussion

**Fig 6:**
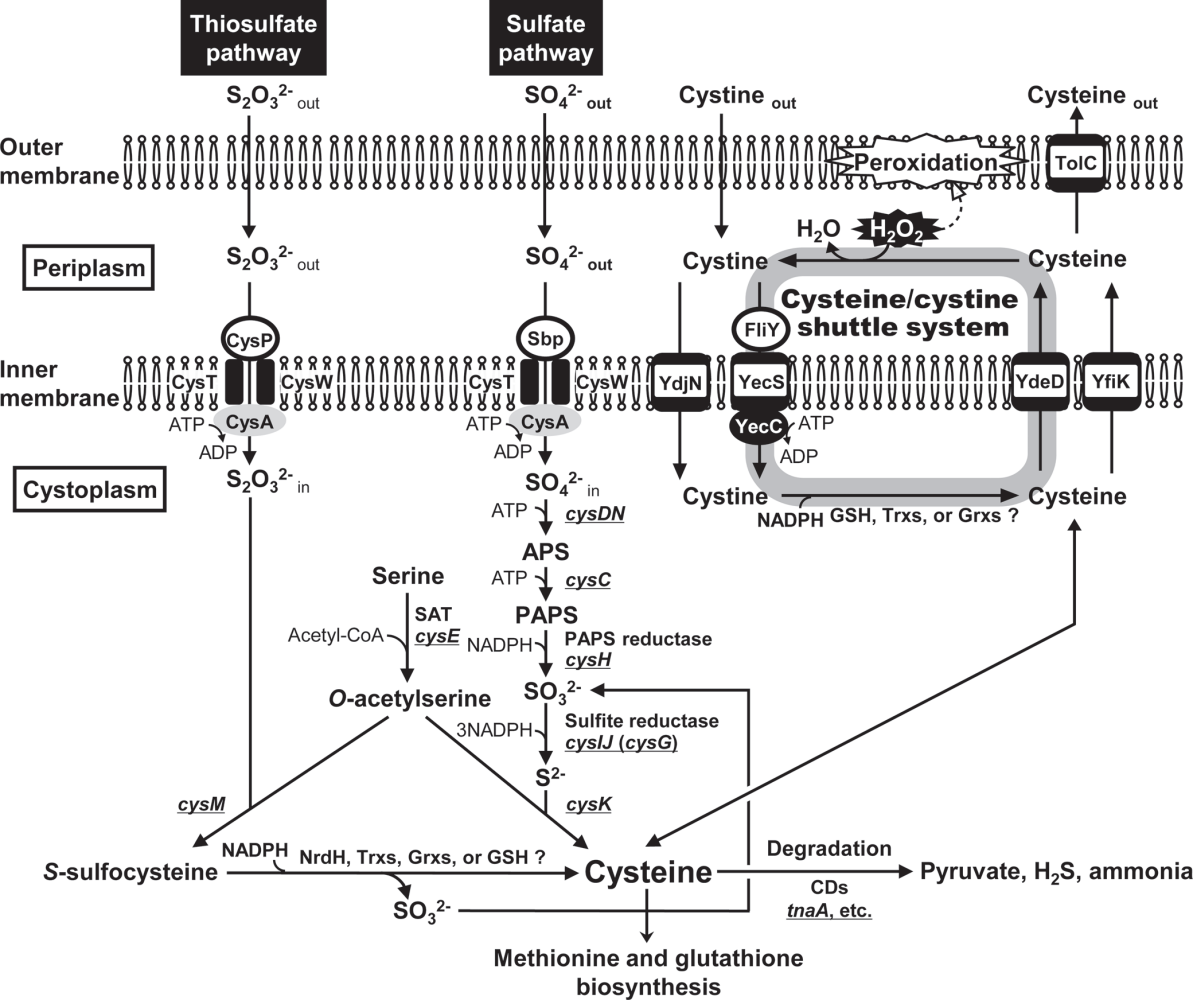
Proposed Cys/CySS shuttle system and overall sulfur metabolism in *E*. *coli*. It is of note that sulfate and thiosulfate utilize common importer CysTWA. GSH, glutathione; Trx, thioredoxin; Grx, glutaredoxin; SAT, serine acetyltransferase; APS, adenosine 5'-phosphosulfate; PAPS, 3'-phosphoadenosine 5'-phosphosulfate; CD, cysteine desulfhydrase.

Leive and Davis [17] have shown that *E*. *coli* has two kinetically identifiable CySS uptake systems: one shared with and competitively inhibited by diaminopimelic acid (DAP), which refer to as the CySS uptake, and the other is more specific. However, a CySS importer in *E*. *coli* remained unknown. We provided evidence here that *E*. *coli* has only two different CySS importers of a low affinity CySS importer YdjN (*K*_m_ = 1.1 μM) and a high affinity ABC CySS importer FliY-YecSC (*K*_m_ = 110 nM). Our results also suggest that the Cys transporter YdeD and the CySS importer FliY-YecSC coordinately function to drive cycling of cytoplasmic Cys and periplasmic CySS. This cycling could be mediated by *de novo* Cys synthesis via assimilation of inorganic sulfur sources such as sulfate or thiosulfate (Fig. 6). Further, the TBARS contents in mutant cells missing FliY and YdjN, or FliY, YdjN and YdeD increased 2.5-fold or 2.2-fold, respectively, even without treatment of H_2_O_2_ (Fig. 5 A and B). In addition, glutathione is a redox-active tripeptide (l-γ-glutamyl-l-cysteinylglycine; GSH) that is present in the cytoplasm of many organisms. It is reported that GSH also exists in the periplasm of *E*. *coli*. We have already reported that hydrogen peroxide tolerance was increased by YdeD overexpression even in the mutant deleted of *gshA* gene, which encodes γ-glutamylcysteine synthetase, a key enzyme in GSH synthesis. This findings suggests that, in contrast to Cys, GSH may not contribute to the hydrogen peroxide resistance of *E*. *coli* cells [5]. These findings led us to propose that Cys/CySS shuttle system provides physiological function in quality control of membrane lipids to avoid its peroxidation under oxidative stress conditions.

The question then is why *E*. *coli* cells possess two CySS importers. It has been reported that *E*. *coli* controls intracellular Cys levels to less than 1 μM due to feedback inhibition of l-serine acetyltransferae by Cys [18, 19], which could be part of the reason for the cytotoxicity of Cys in this organism. In addition, the LysR type transcriptional regulator CysB increases the expression of most of the genes involved in Cys synthesis via sulfur assimilation under sulfur-limited conditions. Indeed, analysis of motif prediction program (MEME) successfully detected the CysB binding motif in the promoter region of *ydjN* as well as those of other known Cys regulon [20]. Thus, we propose that *ydjN* is a new member of Cys regulon. YdjN relatively functions at high concentrations of CySS and can uptake CySS more rapidly than FliY-YecSC does (Table 1). Especially, the *V*_max_ value of YdjN is comparable to that of the wild-type, meaning that almost all CySS uptake occurs via YdjN under CySS rich conditions. Indeed, the disruption of *ydjN* enhanced expression of the genes encoding FliY-YecSC presumably to complement the failure of the CySS uptake ability (Fig. 2D). Consistent with this idea, addition of Cys into the medium decreases the mRNA level of *ydjN* by 30-fold (data not shown). Together with these results, the ability of the low affinity CySS importer, YdjN might play an important role for limited nutrients such as sulfur starvation. In contrast, the high affinity ABC-type CySS importer, FliY-YecSC (*K*_m_ = 110 nM), is inducible in response to H_2_O_2_ (Fig. 4C), but not sulfur starvation. In addition, the CysB binding motif does not exist in the upstream region of the *fliY* or *yecSC* gene. Thus, this system can perform efficient uptake of periplasmically-generated CySS even at low level. These findings suggest that an endogenously produced Cys is in fact exported into the periplasm, where it is oxidized into CySS. These observations are consistent with the proposed role of Cys as a reducing equivalent in the periplasm.

To assess if Cys/CySS shuttle system displays a general antioxidant function in the periplasm, the level of periplasmic H_2_O_2_ in mutants lacking Cys/CySS shuttle system was observed with BES. The introduction of Δ*ydeD*Δ*fliY* into Hpx^—^background cells resulted in accumulation of H_2_O_2_ in the periplasm (Fig. 4A). A question then arises whether the Cys exporter and the CySS importer cooperatively function in Cys/CySS shuttle system? The CySS import back into the cytoplasm via FliY-YecSC is essential for defense of cellular membrane from oxidative stress (Fig. 4B). This result means that both CySS import via FliY-YecSC and Cys export via YdeD function cooperatively. *E*. *coli* cells will again face growth inhibition by the increase in Cys toxicity, if H_2_O_2_ is detoxified by *de novo* synthesized Cys. Thus, this “shuttling” but not “unidirectional exporting” is important and exquisite system for growth, because the system allows cells to scavenge H_2_O_2_ without growth inhibition or without energetic consumption from sulfur assimilation for Cys synthesis.

The dearth of knowledge about the prokaryotic glutathione peroxidases prompted us to analyze the role of Cys/CySS shuttle system in the periplasm to ROS. *E*. *coli* cells lacking CySS transporters or/and Cys transporter showed increased levels of lipid peroxides regardless of the presence or absence of H_2_O_2_ (Fig. 5 A and B). But an increase of protein carbonyl content in these mutants cannot observed (data not shown). These results are suggesting that Cys/CySS shuttle system participates in preventing membrane lipids or controlling the level of membrane peroxidation products. Thus, these findings including our results suggest that the CySS importers and the Cys transporter cooperatively play important roles by supplying a reducing equivalent to the oxidative cellular compartments. It is likely that such an analogous Cys/CySS shuttle system is highly conserved from bacteria to mammals in the inter-membrane and supplies a reducing equivalent to the oxidative cellular compartments.

## Supporting Information

S1 Fig YecC is probably functional ATPase as CySS impoter. Alignment of motif required for ATPase activity of YecC homologs are shown. Walker A, Walker B, and ABC signature motifs are shown with amino acid number of each protein. Non-conserved residues were represented in gray characters. Ec, *Escherichia coli*; Bs *Bacillus subtilis*; Lr, *Lactobacillus reuteri*; Lf, *Lactobacillus fermentum*.
(TIF)

S1 Table Bacterial strains used in this study. (PDF)

S2 Table Oligonucleotides used as primer for real-time PCR. (PDF)

## References

[1] Imlay JA. Pathways of oxidative damage. Annu Rev Microbiol. 2003;57:395–418. 10.1146/annurev.micro.57.030502.090938 14527285

[2] Tani A, Inoue C, Tanaka Y, Yamamoto Y, Kondo H, Hiradate S, et al The crucial role of mitochondrial regulation in adaptive aluminium resistance in *Rhodotorula glutinis* . Microbiology. 2008;154(Pt 11):3437–46. 10.1099/mic.0.2007/016048-0 18957597

[3] Riemer J, Bulleid N, Herrmann JM. Disulfide formation in the ER and mitochondria: two solutions to a common process. Science. 2009;324(5932):1284–7. 10.1126/science.1170653 19498160

[4] Wiriyathanawudhiwong N, Ohtsu I, Li ZD, Mori H, Takagi H. The outer membrane TolC is involved in cysteine tolerance and overproduction in *Escherichia coli* . Appl Microbiol Biotechnol. 2009;81(5):903–13. 10.1007/s00253-008-1686-9 18828007

[5] Ohtsu I, Wiriyathanawudhiwong N, Morigasaki S, Nakatani T, Kadokura H, Takagi H. The L-cysteine/L-cystine shuttle system provides reducing equivalents to the periplasm in *Escherichia coli* . J Biol Chem. 2010;285(23):17479–87. 10.1074/jbc.M109.081356 20351115 PMC2878512

[6] Sambrook J, Fritsch EF, Maniatis T. Molecular Cloning: A Laboratory Manual. 2nd ed. New York: Cold Spring Harbor Laboratory Press; 1989.

[7] Park S, You X, Imlay JA. Substantial DNA damage from submicromolar intracellular hydrogen peroxide detected in Hpx- mutants of *Escherichia coli* . Proc Natl Acad Sci U S A. 2005;102(26):9317–22. 10.1073/pnas.0502051102 15967999 PMC1166606

[8] Baba T, Ara T, Hasegawa M, Takai Y, Okumura Y, Baba M, et al Construction of *Escherichia coli* K-12 in-frame, single-gene knockout mutants: the Keio collection. Mol Syst Biol. 2006;2:2006 0008 10.1038/msb4100050 16738554 PMC1681482

[9] Maness PC, Smolinski S, Blake DM, Huang Z, Wolfrum EJ, Jacoby WA. Bactericidal activity of photocatalytic TiO(2) reaction: toward an understanding of its killing mechanism. Appl Environ Microbiol. 1999;65(9):4094–8. 10473421 10.1128/aem.65.9.4094-4098.1999 PMC99746

[10] Semchyshyn H, Bagnyukova T, Storey K, Lushchak V. Hydrogen peroxide increases the activities of *soxRS* regulon enzymes and the levels of oxidized proteins and lipids in *Escherichia coli* . Cell Biol Int. 2005;29(11):898–902. 10.1016/j.cellbi.2005.08.002 16202627

[11] Lo R, Turner MS, Barry DG, Sreekumar R, Walsh TP, Giffard PM. Cystathionine gamma-lyase is a component of cystine-mediated oxidative defense in *Lactobacillus reuteri* BR11. J Bacteriol. 2009;191(6):1827–37. 10.1128/JB.01553-08 19124577 PMC2648363

[12] Burguiere P, Auger S, Hullo MF, Danchin A, Martin-Verstraete I. Three different systems participate in L-cystine uptake in *Bacillus subtilis* . J Bacteriol. 2004;186(15):4875–84. 10.1128/JB.186.15.4875-4884.2004 15262924 PMC451631

[13] Maeda H, Yamamoto K, Kohno I, Hafsi L, Itoh N, Nakagawa S, et al Design of a practical fluorescent probe for superoxide based on protection-deprotection chemistry of fluoresceins with benzenesulfonyl protecting groups. Chemistry. 2007;13(7):1946–54. 10.1002/chem.200600522 17136791

[14] Maeda H, Matsuno H, Ushida M, Katayama K, Saeki K, Itoh N. 2,4-dinitrobenzenesulfonyl fluoresceins as fluorescent alternatives to Ellman's reagent in thiol-quantification enzyme assays. Angew Chem Int Edit. 2005;44(19):2922–5. 10.1002/anie.200500114 15818626

[15] Maeda H, Fukuyasu Y, Yoshida S, Fukuda M, Saeki K, Matsuno H, et al Fluorescent probes for hydrogen peroxide based on a non-oxidative mechanism. Angew Chem Int Ed Engl. 2004;43(18):2389–91. 10.1002/anie.200452381 15114569

[16] Manchado M, Michán C, Pueyo C. Hydrogen peroxide activates the SoxRS regulon in vivo. J Bacteriol. 2000;182(23):6842–4. 11073934 10.1128/jb.182.23.6842-6844.2000 PMC111432

[17] Leive L, Davis BD. Evidence for a gradient of exogenous and endogenous diaminopimelate in *Escherichia coli* . J Biol Chem. 1965;240(11):4370–6. 5321118

[18] Denk D, Bock A. L-cysteine biosynthesis in *Escherichia coli*: nucleotide sequence and expression of the serine acetyltransferase (*cysE*) gene from the wild-type and a cysteine-excreting mutant. J Gen Microbiol. 1987;133(3):515–25. 3309158 10.1099/00221287-133-3-515

[19] Hindson VJ. Serine acetyltransferase of *Escherichia coli*: substrate specificity and feedback control by cysteine. Biochem J. 2003;375(Pt 3):745–52. 10.1042/BJ20030429 12940772 PMC1223735

[20] Kawano Y, Ohtsu I, Kazuhiro T, Tamakoshi A, Nonaka G, Funahashi E, et al Enhancement of L-cysteine production by disruption of *yciW* in *Escherichia coli* . J Biosci Bioeng. 2015;119: 176–179. 10.1016/j.jbiosc.2014.07.006 25103863

