# Supplementary figures and images for: Uptake of L-cystine via an ABC transporter contributes defense of oxidative stress in the L-cystine export-dependent manner in Escherichia coli
Source: PLoS One. 2015 Apr 2;10(4):e0120619. doi: 10.1371/journal.pone.0120619 (PMC4383340; doi:10.1371/journal.pone.0120619)

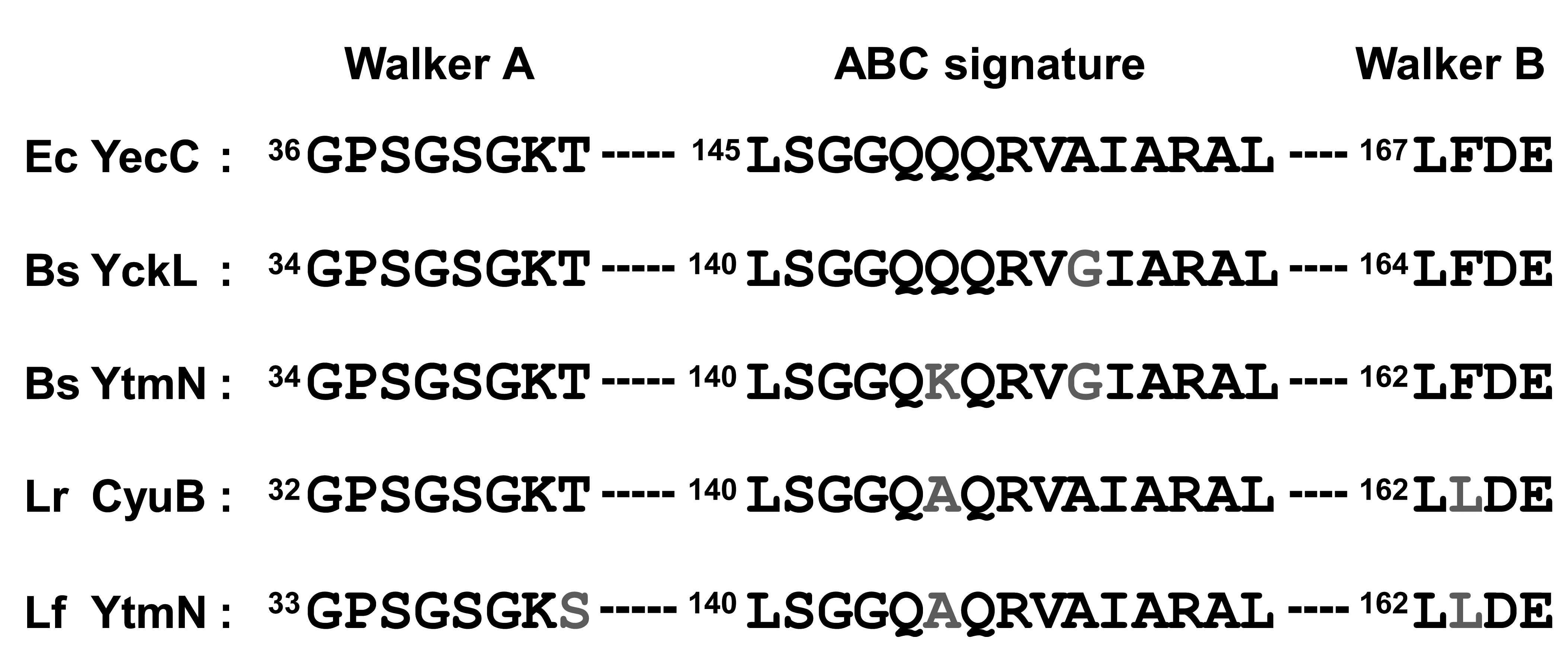

Supplement: S1 Fig — Alignment of motif required for ATPase activity of YecC homologs are shown. Walker A, Walker B, and ABC signature motifs are shown with amino acid number of each protein. Non-conserved residues were represented in gray characters. Ec, Escherichia coli; Bs Bacillus subtilis; Lr, Lactobacillus reuteri; Lf, Lactobacillus fermentum. (TIF) [file pone.0120619.s001.tif]
