# Supplementary material for: Uptake of L-cystine via an ABC transporter contributes defense of oxidative stress in the L-cystine export-dependent manner in Escherichia coli
Source: PLoS One. 2015 Apr 2;10(4):e0120619. doi: 10.1371/journal.pone.0120619 (PMC4383340; doi:10.1371/journal.pone.0120619)
Supplement: S1 Table — (PDF) [file pone.0120619.s002.pdf]

Table S1. Bacterial strains used in this study.

Strain name	Genotype	Source or reference
wild-type	*lacI*^{q}, *rrnB*$_{T14}$ *lacZ*$_{WJ16}$ *hsdR*$_{514}$ *araBAD*$_{AH33}$ *rhaBAD*$_{LD78}$	[7]
Δ*ydeD*	BW25113 *ydeD*::Km^{R}	[7]
Δ*fliY*	BW25113 *fliY*::Km^{R}	[7]
Δ*yecS*	BW25113 *yecS*::Km^{R}	[7]
Δ*yecC*	BW25113 *yecC*::Km^{R}	[7]
Δ*ydjN*	BW25113 *ydjN*::Km^{R}	[7]
Δ*ydjN*Δ*fliY*	BW25113 Δ*fliY* *ydjN*::Km^{R}	This study
Δ*ydjN*Δ*yecS*	BW25113 *yecS*::Km^{R} *ydjN*::Cm^{R}	This study
Δ*ydjN*Δ*yecC*	BW25113 *yecC*::Km^{R} *ydjN*::Cm^{R}	This study
Δ*fliY*Δ*ydeD*	BW25113 Δ*ydeD* *fliY*::Km^{R}	This study
Δ*fliY*Δ*ydjN*Δ*ydeD*	BW25113 Δ*ydeD* *fliY*::Km^{R} *ydjN*::Cm^{R}	This study
Hpx^{-}	MG1655 Δ*ahpCF´ kan::'ahpF* Δ(*katG17*::Tn*10*)1 Δ(*katE12*::Tn*10*)1	[6]
Hpx^{-}Δ*ydeD*Δ*fliY*	Hpx^{-} Δ*ydeD* *fliY*::Cm^{R}	This study

Km^{R}, kanamycin resistant; Cm^{R}, chloramphenicol resistant.
