# Supplementary material for: Uptake of L-cystine via an ABC transporter contributes defense of oxidative stress in the L-cystine export-dependent manner in Escherichia coli
Source: PLoS One. 2015 Apr 2;10(4):e0120619. doi: 10.1371/journal.pone.0120619 (PMC4383340; doi:10.1371/journal.pone.0120619)
Supplement: S2 Table — (PDF) [file pone.0120619.s003.pdf]

Table S2. Oligonucleotides used as primer for real-time PCR.

Name	Sequence (5'-3')
YdeD-RT-FW	CGATGCTCGGCTTTATGTTGA
YdeD-RT-RV	TTGCCACACGCCCAACT
KatG-RT-FW	GCCCGTGCCTGGTTCA
KatG-RT-RV	GGCCCGATGTAGCGAGATT
SodA-RT-FW	CCGCTGATGGGTGAAGCTAT
SodA-RT-RV	CACATCCAGGCCCATAATCG
YdjN-RT-FW	CCCTGGTGGCGCTGTTAA
YdjN-RT-RV	CGTACGGCCCATGTCGATA
FliY-RT-FW	GCTGGTAGGGCTGGAAGGA
FliY-RT-RV	TTGCCGTCATCTCCCTGAA
YecC-RT-FW	CAGCTGGCGCAGGAAAA
YecC-RT-RV	CGGGCAAAGCTCATTTCGT
YecS-RT-FW	GCCTATGCTGCCGAAACG
YecS-RT-RV	CCGCTTCCCACTGACCTTTA
DcyD-RT-FW	TGGGCGGCAATAAATTACGT
DcyD-RT-RV	GCACCTTCACGCAGAGCAT

FW, forward; RV, reverse.
